# Effects of yoga on impulsivity in patients with and without mental disorders: a systematic review

**DOI:** 10.1186/s12888-024-05608-3

**Published:** 2024-04-09

**Authors:** Yuri de Castro Machado, Mariana Oliveira, Jogiely Larissa Ferreira Lima, Hemant Bhargav, Shivarama Varambally, Débora Marques de Miranda, Marco Aurélio Romano-Silva

**Affiliations:** 1 Universidade Federal de Minas Gerais, Belo Horizonte, Brazil; 2 Faculdade de Ensino Superior da Amazônia Reunida, Redenção, Brazil; 3 National Institute of Mental Health and Neurosciences, Bengaluru, India

**Keywords:** Yoga, Impulsivity, Mental Disorders, Mental Health

## Abstract

**Background:**

Yoga can be used as a complementary intervention to conventional treatments, whether pharmacological or non-pharmacological. Sustained practice of yoga can generate a series of benefits for individuals' quality of life and improve their physical fitness.

**Objective:**

To investigate the potential effects of yoga as an adjunct intervention in conditions involving impulse control issues, such as attention deficit hyperactivity disorder (ADHD), borderline personality disorder, bipolar affective disorder, and substance use disorders.

**Methods:**

We performed a systematic review of placebo-controlled, randomized trials of yoga in patients with impulsivity. PubMed, Web of Science, and Science Direct databases were searched for trials published up to January, 2023. Data were extracted from published reports and quality assessment was performed per Cochrane recommendations.

**Results:**

Out of 277 database results, 6 RCT were included in this systematic review. To assess the level of attention and impulsiveness, the following scales were analyzed: Barratt Impulsiveness, UPPS-P Impulsive Behavior scale, Conners’ Continuous Performance Test IIª and Conners’ Parent Rating Scale–Revised: Long.

**Conclusions:**

Yoga didn’t have a significant improvement in impulsivity when compared to placebo. There are many tools to assess impulsivity, but they mean different concepts and domains consisting in a weakness on comparison of yoga effects.

**PROSPERO registration:**

CRD42023389088.

**Supplementary Information:**

The online version contains supplementary material available at 10.1186/s12888-024-05608-3.

## Introduction

Impulsivity is an inter-individual characteristic with an impact on many psychiatric disorders, such as some personality disorders, eating disorders, substance use disorders, and self-injurious behavior. Individuals who express more impulsivity traits are more likely to have accidents, have addiction-related disorders, and worse coping under stressful conditions [1]. In conditions of substance use, impulsivity affects the course of treatment and may be related to a lack of forethought and negative urgency, leading to a poor response to psychotherapy. However, in the case of a decrease in two impulsivity traits, negative urgency and novelty seeking appear to decrease slightly, having a small favorable impact without treatment [2].

The practice of Yoga has a probable millennial origin and was built on a complex set of mind–body and philosophical elements that involves mindful practice of physical postures, breathing techniques, meditations, relaxation techniques and certain lifestyle principles, essentially aiming to offer man a path of living the existence with purpose and fullness. As it constitutes a system of multifactorial practices, throughout history several modalities have been created to improve its execution. In general, they are all based on the following pillars: breathing technique and development of self-awareness; posture training including stretching exercises and specific postures (asana); body relaxation activities and meditative practices focused on cognitive control and attention in the present. In western cultures, postures, self-awareness, and meditation are taught more often, leaving behind part of the ethical and moral code related to philosophy [3]. Sessions can be carried out following two main strands: long-term and low-frequency practices or short-term and high-intensity practices. An example of the second strand is the Vipassana program in which individuals practice yoga (in the form of disciplined mindful attention to the physical sensations, in a steady pose) for 10 days, spending an average of 11 h a day.

Despite the methodological limitations in currently available scientific literature, several clinical trials and meta-analyses demonstrate clinical utility of yoga as a complementary intervention to conventional treatments, whether pharmacological or non-pharmacological. Sustained practice of yoga can generate a series of benefits for individuals' quality of life, improve their physical fitness [4] and also their emotional state [5, 6]. The use of objective scientific tools such as electroencephalogram (EEG) in studies allowed the demonstration of possible physiological changes due to yoga in the body. A study demonstrated that there was a reduction in oxygen consumption following a yoga-based relaxation technique [7]. Similarly, another study demonstrated regulation of brain activity following yoga [8]. Understanding the relationship between yoga activity and brain functioning, especially related to cognition, is of great importance for clinical conditions associated with impulsivity. A specific work demonstrated that as compared to control group (yoga naive subjects), yoga practitioners showed greater activation of ventrolateral prefrontal cortices (vlPFC) during stroop task. vlPFC is associated with a better executive-dependent capacity and ability to reduce emotional interference during competing cognitive demands [9].

These emerging scientific evidences, along with absence of significant reports on the possible adverse effects [10], and its low cost of implementation make yoga a potentially useful adjunct treatment in conditions involving impulse control issues [11, 12]. Thus, current systematic review aimed at investigating the potential effects of yoga as an adjunct intervention in conditions involving impulse control issues, such as attention deficit hyperactivity disorder (ADHD), borderline personality disorder, bipolar affective disorder, and substance use disorders.

## Methods

**Fig. 1:**
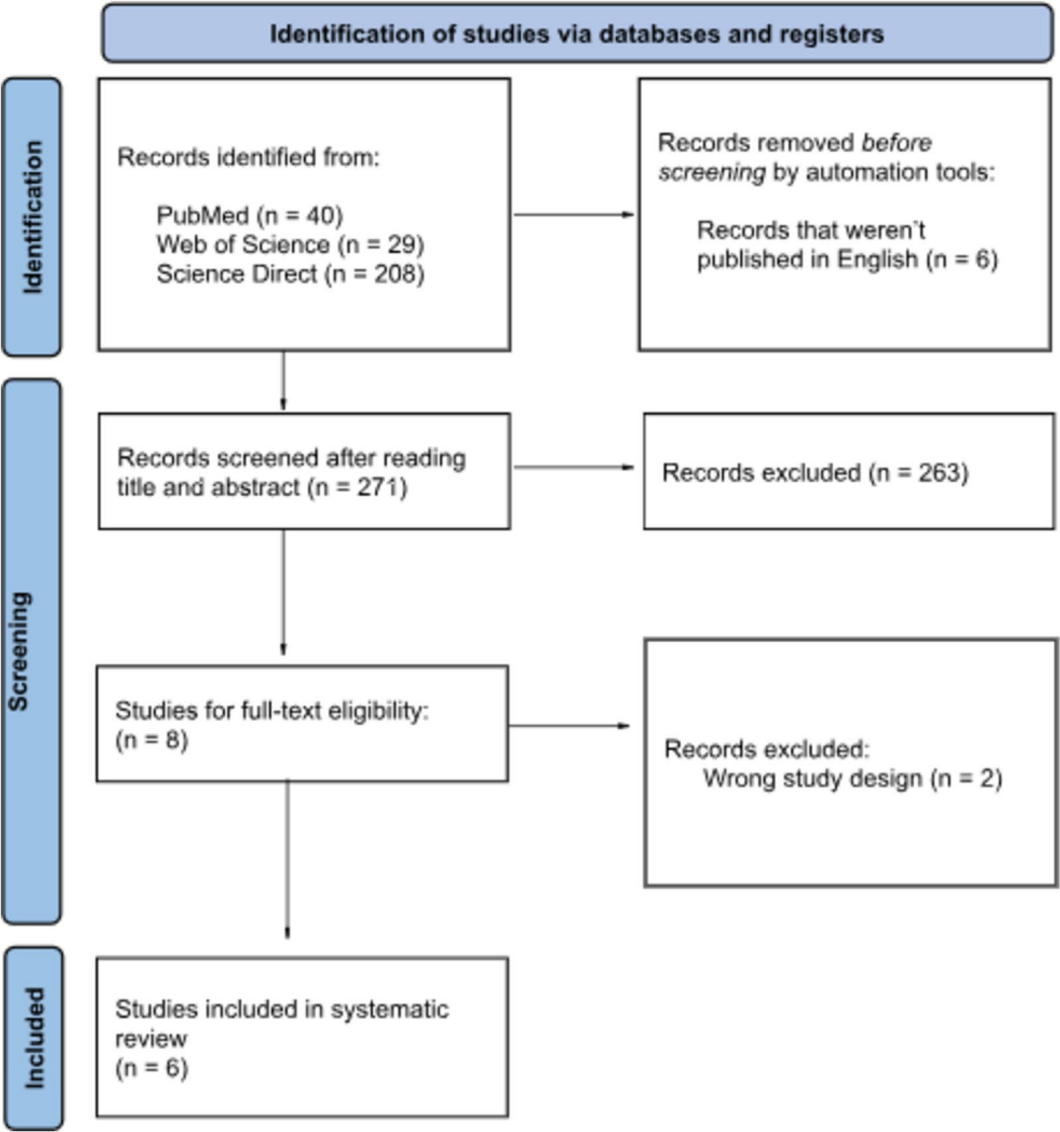
Screening of the articles following the PRISMA Flowchart

This systematic review followed the Preferred Reporting Items for Systematic Reviews and Meta-Analysis (PRISMA) Statement [13], as shown in Fig. 1. The review protocol was registered in the International Prospective Register of Systematic Reviews (PROSPERO, registration number (CRD42023389088).

### Elegibility criteria

Inclusion in this systematic review was restricted to studies that met all the following eligibility criteria: (1) randomized trials or non-randomized cohorts; (2) those in patients with impulsivity (ADHD with impulsivity and Non-ADHD; borderline disorder; bipolar disorder; medication users; tabagists; addiction) doing yoga; (3) those with control group (placebo or waitlist); and (4) those reporting any of the outcomes of interest (improvement of impulsivity with objective and subjective scales). Exclusion criteria was based on (1) studies without a control group.

### Search strategy

We systematically searched PubMed, Scopus, and Science Direct in January 2023 using the following search strategy: (impulsivity OR impulsive behaviors OR impulsivities) AND yoga. The search was restricted to articles published in English and the period of publication was not delimited.

### Data extraction

Two authors (MO and JL) independently assessed the articles for inclusion and in case of disagreement, a third evaluator deliberated. This process was executed through Rayyan.ai—Intelligent Systematic Review. After the triage phase, both authors (MO and JL) independently extracted the data after predefined search criteria and quality assessment.

### Quality assessment

Quality assessment of RCTs was performed with Cochrane's tool for assessing bias in randomized trials (ROB-2) where in studies are scored as high, low, or some concerns of bias in 5 domains: randomization process, deviations from intended interventions, missing outcome data, measurement of the outcomes, and reporting [14]. Publication bias was investigated by funnel-plot analysis of point estimates according to study weights and by Egger’s regression test [15].

## Results

### Description of studies

After the screening process, six studies met the inclusion criteria. The study characteristics are summarized in Table 1. There were 549 participants across the results, with 36.4% overall female; only one study engaged exclusively male participants. Only one study had a randomized crossover design [16]. Two studies used groups of children diagnosed with ADHD [16, 17]; two studies analyzed prison population [18, 19]; one study selected groups of meditators expertises [20] and one study analyzed a population of 8 to13 year old boys [21]. The control group ranged from waitlist, non-meditators, cooperative activities and physical education among the studies.

**Table 1:** Characteristics of the selected studies

	Kerekes 2017 [19]	Barrós-loscertales 2021 [20]	Jensen & Kenny 2004 [21]	Cohen 2018 [16]	Butzer 2017 [17]	Bilderbeck 2013 [18]
Nº of patients	152	46	19	23	211	167
Population	Prison population	Meditators expertises	8–13 years boys	Children diagnosed with ADHD	Children with 4 or more attention- deficit hyperactivity disorder (ADHD) symptoms	Prison population
Intervention	Combination of asanas (yoga postures), breathing exercises, deep relaxation, and meditation	To achieve the state of mental silence or thoughtless awareness, where thoughts are either suppressed or substantially reduced	Twenty weekly 1-h yoga instructional group sessions	30-min group yoga sessions were held twice a week plus 30-min yoga protocol themes according to instructions in a yoga DVD	32-session of the Kripalu Yoga	Classes were held in a quiet room and consisted of a standardised set of hatha yoga postures and stretches. To complement the poses, the final 10–20 min of each class were spent doing relaxation
Session time	90 min	84 min	1 h	30 min	45 min	2 h
Nº of sessions	10	-	20	12	32	10
Control group	Waitlist	Non-meditators	Cooperative activities	Waitlist	physical education-as-usual	Prisioners without experience on meditation
Median follow-up	10 weeks	-	20 weeks	6 weeks	6 months	10 weeks

### Intervention characteristics

All the studies used yoga techniques that involved respiratory training, postures and relaxation [16–22]. Hatha yoga was used by one study [18] and one study tested the program Kripalu Yoga [17]. All the yoga sessions had more than 30 min of duration; and all of the studies had 10 sessions [20]. Two studies had a 10-week median follow-up; one study didn’t mention it [20].

### Intervention measurements

To assess the level of attention and impulsiveness, the following scales were applied: The CCPT-II [18, 20], The UPPS-P Impulsive Behavior scale [17], ADHD Rating Scale-IV [16], Conners’ Continuous Performance Test IIª [19] and Conners’ Parent Rating Scale–Revised:Long [21].

Conners’ CPT II is a computerized test that measures attention, impulsivity and vigilance. After 10 weeks of yoga, The Conners’ CPT II identified that the yoga group had significantly fewer errors of commissions (Incorrect responses to non-targets), increased hit reaction time (Response Speed) and better detectability compared to the control group [19]. Furthermore, participants in the yoga group demonstrated improved performance in a cognitive-behavioral task compared to the control group [19].

Butzer et al. (2017) [17] used the UPPS-P to predict the results of the relation between yoga and impulsivity. About negative urgency, positive urgency and sensation seeking, males reported significantly higher levels than females. Post analyzing the lack of premeditation, females in the yoga group reported a significant increase between one week pre-intervention and one-week post-intervention, there was a significant decrease from one-week post-intervention and 6 months post-intervention, though males in yoga group did not report significant changes in lack of premeditation. Conversely, females in a control group reported a significant increase in lack of premeditation between one-week post-intervention and one-year post-intervention, though males in the control group did not report significant changes in lack of premeditation.

The Sahaja Yoga meditation showed an increased self-control score in the BIS-11 compared to the control participants, with a total BIS-11 score of meditators equal 61.91 and control group 58.83 [20].

### Subgroup characteristics

Baseline subgroup characteristics of each study are detailed in Table 2. Of the studies that reported gender (*n* = 6), approximately 63,57% (*n* = 349) of the participants were male and 36,43% (*n* = 200) were female. The median participants' mean age ranged from 10.63 to 46.5 on the yoga group, and 9.35 to 46.9 on the control group. One study did not report ethnicity (Kerekes et al., 2017) [19]. In the remaining studies, 271 participants were white/Caucasian, 27 black participants, 70 Chinese/Asian descendent participants, 21 mixed participants and 5 unknowns. Furthermore, two studies reported economic conditions, prevailing low-middle- and low-income conditions.

**Table 2:** Baseline subgroup characteristics

	Kerekes 2017 [19]	Barrós-loscertales 2021 [20]	Jensen & kenny 2014 [21]	Cohen 2018 [16]	Butzer 2017 [17]	Bilderbeck 2013 [18]
**Age (mean)**	I = 36.4 C = 34.9	I = 46.5 C = 46.9	I = 10.63 C = 9.35	I = 52 ± 7 C = 46 ± 10	12.64	37.38
Gender						
**Male**	133	12	19	15	77	93
Female	19	34	0	8	132	7
**Race**						
White	-	46 (white Caucasian)	18	10	117	80
**Black**	-	-	-	9	9	9
Brown	-	-	-	-	-	-
**Chinese/ Asian**	-	-	1	1	61	7
Mixed	-	-	-	2	16	3
**Unknown**	-	-	-	1	4	-
Economic condition	-	-	Low-middle	-	Graduation rate was 98.9%, and 34.5% of students were considered low income	-
**Schooling**	-	3.78 (yoga group) 4.04 (control group)	-	First year (*n* = 14) Second year (*n* = 3) Third year (*n* = 3) Fourth year (*n* = 2)	Grades 7 through 12	-
ADHD	-	-	All (*n* = 19)	2	-	-
**PTSD**	-	-	-	-	-	-
Addiction	-	-	-	-	-	-

Three studies reported schooling. Participants’ mean schooling age ranged from 3 to 12. About mental disorders, two studies reported participants with ADHD [16, 21] and didn't report post trauma stress disorder or addiction.

### Quality assessment

The funnel plot (supplementary Fig. 1) and the Egger’s test (beta1 = -2.88.; SE of beta1 = 2.376; t = -1.21, P >|t|= 0.3490) did not show statistically significant evidence of publication bias. The quality of evidence concerning the impact of yoga on impulsivity was appraised as high, as indicated by the downgrading criteria applied to the analyzed parameters (Table 3).

**Table 3:** Quality assessment of the studies using ROB-2

Study	Bias from randomization process	Bias due to deviations from intended interventions	Bias due to missing outcome data	Bias in measurement of the outcomes	Bias in selection of the reported result	Overall risk of bias
Kerekes et al. 2017 [19]	Low	Some concerns	Low	High	Low	High
Jensen & Kenny, 2014 [21]	Some concerns	Low	High	Some concerns	Some concerns	High
Cohen et al. 2018 [16]	Low	Low	High	Some concerns	Low	Some concerns
Barrós-Loscertales et al. 2021 [20]	Low	Some concerns	Low	Low	Some concerns	Some concerns
Butzer et al. 2017 [17]	High	Some concerns	Low	Some concerns	Some concerns	High
Bilderbeck et al. 2013 [18]	Low	Some concerns	Low	Some concerns	Some concerns	High

## Discussion

The review protocol included only RCTs, the need for comparison groups and trials comparing intervention strategies made a revision process restrictive, but with consistent results and impressions. Few studies have many different strategies of Yoga, with different amounts of sessions, and strategies to measure different domains of impulsivity. So, there is moderate heterogeneity in a few studies to have confidence to say if it is advantageous to improve impulsivity.

Considering Yoga has many applications and varied practices commonly including a lifestyle change. Still, it fills the inclusion criteria since it has respiratory training, postures, and relaxation, it might not be related to changes in daily life since the individuals were evaluated after 10–32 sessions. Findings on impulsivity should be better identified in longer-term studies in which those observed practice yoga and implement lifestyle changes. With few weekly hours of meditation and breath exercises only, the expected effects should be heterogeneous.

Anyway, Yoga as a holistic therapy using varied practices aiming to relax the body, control breath and calm down the mind might result in enhanced health. Yoga as an ability to turn the mind to sustained attention to an object and avoid distractions might be the most interesting to reach good results for impulsivity control. The principles and values might be compatible with an individual's improvement in impulsivity. Yoga as an expression of integrative medicine allows a practice of personalized medicine, a safe environment, a cost-effective treatment, and a non-maleficence practice [23].

It is noteworthy that impulsivity has been prone to confusion and ambiguity in both terminology and conceptual understanding. Impulsivity is not a singular trait but rather a multifaceted characteristic influenced by different psychological and neural mechanisms. Impulsive behavior can be associated with heightened motivation as well as reduced motivation (referred to as 'apathy'). It can reflect inadequate information processing or a failure to regulate responses. The Barratt Impulsiveness Scale 11 (BIS-11) captures this heterogeneity through three different dimensions: motor (action without thinking), cognitive (quick cognitive decision-making), and non-planning (decrease in orientation towards future) [24, 25]. However, only one of the studies selected in our review used BIS-11 as a measurement of impulsivity [20].

The Conners’ CPT II used by Bilderbeck et al. (2013) [18] can provide valuable insights into impulsivity, however, it has some important disadvantages, such as limited assessment scope, lack of contextual factors, reliance on self-report measures, and interpretation challenges. Interpreting the results of the CPT II requires expertise and knowledge of the test's psychometric properties. Misinterpretation of scores or failure to consider individual differences can lead to inaccurate assessments of impulsivity [26, 27].

Butzer et al. (2017) [17] was the only study that used the UPPS-P Impulsive Behavior scale, a 59-item self-report scale which is a revised version of the original UPPS created by Whiteside and Lynam (2001) [28]. It reveals five distinct facets of impulsivity. These facets include sensation seeking, which reflects a tendency to seek novel and thrilling experiences; lack of premeditation, which involves a disregard for the consequences of actions; and lack of perseverance, which relates to difficulty in maintaining focus on long, boring, or challenging tasks. Additionally, the traits of negative urgency entail impulsive actions during intense negative moods, while positive urgency involves impulsive actions during intense positive moods [29, 30].

Jensen and Kenny (2014) [21] used the long version of The Conners’ Parent Rating Scale-Revised (CPRS-R: long), which is the parent form of the Conners’ Rating Scales-Revised (CRS-R) [31]. It is a parent-report measure that assesses children’s problem behaviors, particularly symptoms of ADH) and related disorders (including oppositional defiant disorder and conduct disorder). However, the CPRS-R has some disadvantages when used to assess impulsivity. One of them is that it relies on the parent’s subjective report of their child’s behavior, which may not always be accurate [32]. Another disadvantage is that it does not have formal reliability and validity scales [31]. Instead, the manual recommends that the mental health professional using the CPRS-R examine the protocol for random responding by assessing for an overabundance of one particular answer and zigzag patterns [33].

The Conners' Continuous Performance Test-II [34], used by Kerekes et al. (2017) [19], is a visual assessment tool designed to evaluate attention and measure the response inhibition component of executive control. Its purpose is to provide a reliable and objective measure for the assessment of conditions such as ADHD and other neurological disorders. By utilizing this test, clinicians aim to incorporate standardized and objective evaluations into their diagnostic processes, enhancing the accuracy of assessments for these conditions. It also is a widely used measure of attention and impulsivity. However, only a minimal amount is known about its reliability. Findings indicated that the CCPT-II had strong internal consistency, adequate test–retest reliability for commission errors and response time, poor test–retest reliability for omission errors, and practice effects for omission and commission errors. The CCPT-II was largely unrelated to the Behavior Rating Inventory of Executive Function for Adults (BRIEF-A), Stroop Color and Word Test, and State-Trait Personality Inventory (STPI) [35]. There are many tools to assess impulsivity, but they mean different concepts and domains consisting of weakness in comparison to yoga effects.

### Limitations

Our study has some important limitations. Firstly, the limited number of available studies and the high heterogeneity among them present challenges in drawing definitive conclusions. The diverse strategies of yoga, varying session durations, and different measures of impulsivity across studies contribute to the overall heterogeneity. Consequently, caution is warranted in interpreting the results due to the potential influence of these factors. The evaluation of individuals after a relatively short duration of yoga practice may not capture the full potential effects, and longer-term studies are needed to assess sustained impacts. Moreover, the measurement tools employed in the included studies have their own limitations, such as limited scope, reliance on self-report measures, and challenges in interpretation.

## Conclusions

The results and impressions derived from the included studies were consistent, despite the moderate heterogeneity observed in a few studies. It is important to note that the studies encompassed a wide range of yoga strategies, session durations, and measures of impulsivity, leading to moderate heterogeneity and reducing confidence in drawing definitive conclusions regarding the effectiveness of yoga in improving impulsivity.

The holistic nature of yoga, which encompasses varied practices aiming to relax the body, control breath, and calm the mind, holds potential for enhancing overall health. The ability of yoga to cultivate sustained attention and reduce distractions may be particularly relevant for impulsivity control. The principles and values of yoga align with an individual's improvement in impulsivity, making it a compatible approach. Impulsivity, however, is a multifaceted characteristic influenced by various psychological and neural mechanisms, which has led to confusion and ambiguity in its terminology and conceptual understanding.

Future research should focus on longer-term studies that evaluate sustained impacts and incorporate comprehensive assessments of impulsivity. Despite its limitations, yoga as a holistic therapy shows promise in promoting overall well-being, and its integrative approach aligns with the principles of personalized medicine.

## Supplementary Information

**Supplementary Material 1. **

## Data Availability

All data generated or analyzed during this study are included in this published article and its supplementary information files.

## Abbreviations

ADHD: Attention deficit hyperactivity disorder
BIS-11: Barratt Impulsiveness Scale-11
BRIEF-A: Behavior Rating Inventory of Executive Function for Adults
CPRS-R-long: Conners’ Parent Rating Scale – Revised
CPT II: Continuous Performance Test IIª
EEG: Electroencephalogram
PRISMA: Preferred Reporting Items for Systematic Reviews and Meta-Analysis
RCT: Randomized controlled trial
ROB-2: Cochrane's tool for assessing bias in randomized trials
STPI: State-Trait Personality Inventory
vlPFC: Ventrolateral prefrontal cortices
