# Correction: Effects of yoga on impulsivity in patients with and without mental disorders: a systematic review

**DOI:** 10.1186/s12888-024-05976-w

**Published:** 2024-11-01

**Authors:** Yuri de Castro Machado, Mariana Oliveira, Jogiely Larissa Ferreira Lima, Hemant Bhargav, Shivarama Varambally, Débora Marques de Miranda, Marco Aurélio Romano-Silva

**Affiliations:** 1 Universidade Federal de Minas Gerais, Belo Horizonte, Brazil; 2 Faculdade de Ensino Superior da Amazônia Reunida, Redenção, Brazil; 3 National Institute of Mental Health and Neurosciences, Bengaluru, India

**Correction: BMC Psychiatry (2024) 24:267**

10.1186/s12888-024-05608-3

Following publication of the original article [1], the authors would like to correct the sessions under the heading intervention characteristics, the measurement tool and the numbering of in-text citation. Throughout the article the numbering of in-text citation has been corrected from [20] to [19].

The sentence under the heading Intervention characteristics currently reads: All the yoga sessions had more than 30 min of duration; and all of the studies but one had more than 10 sessions [21].

The sentence under the heading Intervention characteristics should read: All the yoga sessions had more than 30 min of duration; and all of the studies had 10 sessions [21].

The incorrect measurement tool is: Barratt Impulsiveness Scale.

The correct measurement tool is: CCPT-II.

The original article [1] has been corrected.
