# Supplementary figures and images for: Effects of yoga on impulsivity in patients with and without mental disorders: a systematic review
Source: BMC Psychiatry. 2024 Apr 9;24:267. doi: 10.1186/s12888-024-05608-3 (PMC11003078; doi:10.1186/s12888-024-05608-3)

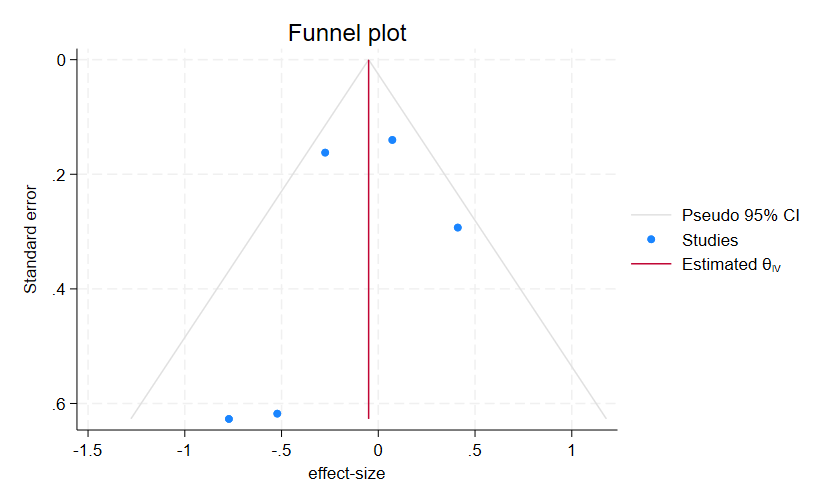

Supplement: Supplementary file 1 — Supplementary Material 1. [file 12888_2024_5608_MOESM1_ESM.png]
